# Linking drought indices to atmospheric circulation in Svalbard, in the Atlantic sector of the High Arctic

**DOI:** 10.1038/s41598-024-51869-z

**Published:** 2024-01-25

**Authors:** Krzysztof Migała, Ewa Łupikasza, Marzena Osuch, Magdalena Opała-Owczarek, Piotr Owczarek

**Affiliations:** 1 Institute of Geography and Regional Development, University of Wroclaw, Pl. Uniwersytecki 1, 50-138 Wroclaw, Poland; 2 Institute of Earth Sciences, University of Silesia in Katowice, ul. Będzińska 60, 41-200 Sosnowiec, Poland; 3 Institute of Geophysics, Polish Academy of Sciences, 64 Księcia Janusza Str., 01-452 Warsaw, Poland

**Keywords:** Atmospheric science, Atmospheric dynamics, Climate sciences, Environmental sciences, Hydrology

## Abstract

Based on long-term climatological data from Ny-Ålesund, Svalbard Airport—Longyearbyen and the Polish Polar Station at Hornsund, we undertook an analysis of drought indices on Spitsbergen Island, Svalbard, for the period 1979–2019. The features and causes of spatiotemporal variability of atmospheric drought in Svalbard were identified, as expressed by the standardized precipitation evapotranspiration index (SPEI). There were several-year periods with SPEI indicating the dominance of drought or wet conditions. The long-term variability in the annual and half-year (May–October) SPEI values showed a prevalence of droughts in the 1980s and the first decade of the twenty-first century, while wet seasons were frequent in the 1990s and in the second decade of the twenty-first century. The seasonal SPEIs were characteristic of interannual variability. In MAM and JJA, droughts were more frequent after 2000; during SON and DJF of the same period, the frequency of wet seasons increased. The most remarkable changes in the scale of the entire research period occurred in autumn when negative values of SPEI occurred more often in the first part of the period, and positive values dominated in the last 20 years. The long-term pattern of the variables in consecutive seasons between 1979 and 2019 indicates relationships between the SPEI and anomalies of precipitable water and somewhat weaker relationships with anomalies of sea level pressure. The three stations are located at distances of more than 200 km from each other in the northern (Ny-Ålesund), central (Longyearbyen) and southern parts of Svalbard (Hornsund), and the most extreme values of drought conditions depended on the atmospheric circulation which could have been modified by local conditions thus droughts developed under various circulation types depending on the station. However, some similarities were identified in the atmospheric circulation patterns favouring drought conditions at Ny-Ålesund and Hornsund, both having more maritime climates than Longyearbyen. Extremely dry seasons were favoured by anticyclonic conditions, particularly a high-pressure ridge (type Ka) centred over Svalbard, air advection from the eastern sector under an influence of cyclone and negative precipitable water anomalies. During wet seasons anomalies of precipitable water were positive and cyclonic conditions dominated. These results were corroborated by the frequency of regional circulation types during JJA and DJF with the lowest and highest values of SPEI.

## Introduction

Feedback mechanisms are causing Arctic amplification, a phenomenon manifested by unprecedented increases in air temperature and total liquid precipitation in polar regions compared with temperate and tropical latitudes^1–3^. These changes are likely to continue in the future, increasing both air temperatures and precipitation amounts^4,5^, facilitating changes in weather and climate-based drivers of glacier recession and thinning^6,7^, permafrost degradation and defragmentation^8–10^, and exacerbating the ecological risk to the whole ecosystem^11–13^.

The frequency and range of extreme climate events are thought to be significant drivers of environmental changes^14^. Extreme events in the Arctic, such as abnormally dry conditions (drought) and abnormally wet (pluvial) ones, are also significantly influencing fragile polar ecosystems. However, their environmental effects in the warm/growing season are different from those in the winter season. Zeng et al.^15^ studied variations in drought during growing seasons in the Northern Hemisphere and found that the duration and frequency of droughts decreased considerably from 1998 to 2015 and that wetting trends were located mainly in high-latitude areas. However, extreme climate events in the Arctic fluctuate and occur alternately^16–19^.

In recent years, research has indicated heterogeneity in the responses of vegetation to climate change in the Arctic^20^. While many Arctic regions have become greener since the 1980s, reflecting the positive response of tundra shrub species to warming and an increase in plant growth, satellite data have shown a decrease in plant productivity in many areas since the early 2000s^21,22^. The number of sites with spectral browning in satellite studies is increasing^23^, which tallies with regional field studies indicating recent declines in shrub growth due to drought stress. The role of precipitation has become increasingly important in recent years, as described for Greenland^24,25^, southern Spitsbergen^26^, Bear Island^13^, Iceland^27^ and Siberia^28^. It should be noted that severe droughts recorded in the Arctic have not only led to tundra browning and reduced productivity but have also resulted in the mortality of species^29,30^. On the other hand, non-drought conditions, i.e. long-lasting, heavy rain events and increased summer precipitation influence hydrological processes and the soil’s thermal regime and stimulate permafrost thaw^31^. Increased precipitation leads to a higher solifluction rate and greater mass movement activity, especially debris flow events^32–34^. Negative precipitation anomalies in winter reduce the snow cover depth and diminish the snow water equivalent (SWE), which may further bring about a negative annual mass balance of the glaciers. On the other hand, positive winter precipitation anomalies have the reverse effect, i.e. increased snow accumulation, higher SWE values and a positive mass balance. Moreover, thicker snow cover in non-glaciated areas increases the avalanche risk and delays the onset of growing seasons and ground thaw^35–39^.

Serreze et al.^40^ stated that extreme events in Spitsbergen tend to occur when the region is influenced by a trough of low sea level pressure extending from the south-west, but that some of the largest precipitation events may be associated with a 500 hPa geopotential height anomaly (positive over the Barents Sea and negative over Greenland) and with positive precipitable water anomalies with a stream extending perhaps thousands of kilometres southwards into the subtropical Atlantic. This statement leads one to assume that wet conditions expressed as drought indices are also related to geopotential height and precipitable water anomalies over the North Atlantic. Thus, the hypothesis to be examined here is that, in contrast to the conditions for extreme precipitation given by Serreze et al.^40^, a deficit of atmospheric water and periods of dryness identified by negative drought indices can also be explained by factors related to the distribution of the baric field over the North Atlantic and regional circulation types.

This paper aims to identify the spatiotemporal variability of atmospheric drought impacting ecosystems in Svalbard and also the atmospheric circulation patterns impacting wet and dry conditions (positive/negative drought indices) in the Atlantic sector of the High Arctic, as represented by Svalbard.

## Area, data and methods

The deficit or excess of water is described by the SPEI (Standardized Precipitation Evapotranspiration Index) in accordance with WMO recommendations in relation to drought indices^41^. The SPEI, which is a unitless index, was developed by Vicente-Serrano et al.^42^ and has been applied in numerous studies^43–45^. It is calculated by normalizing the climatic water balance (precipitation minus potential evapotranspiration) time series.

Based on the long-term climatological data from Ny-Ålesund (NyA), Longyearbyen—Svalbard Airport (LYR) and Hornsund—Polish Polar Station (HOR), we undertook an analysis of drought indices on West Spitsbergen Island, Svalbard, for the period 1979–2019. The data for Ny-Ålesund and Longyearbyen were obtained from the Norwegian Centre for Climate Services (https://seklima.met.no), which provides a quality-controlled series. Despite this, these series had been previously analysed for their quality and were found to be homogeneous, despite the need to correct the air temperature in Ny-Ålesund^46–48^. The data for Hornsund was retrieved from the database published by Wawrzyniak and Osuch^49,50^, which also includes a homogenized data series. The measurements of Arctic precipitation are nevertheless encumbered with a high level of uncertainty owing to wind-induced undercatch, which differs depending on the precipitation phase, wind speed and the type of rain gauge. The undercatch errors for solid precipitation can range from 20 to 50% in windy conditions^51^.

**Figure 1:**
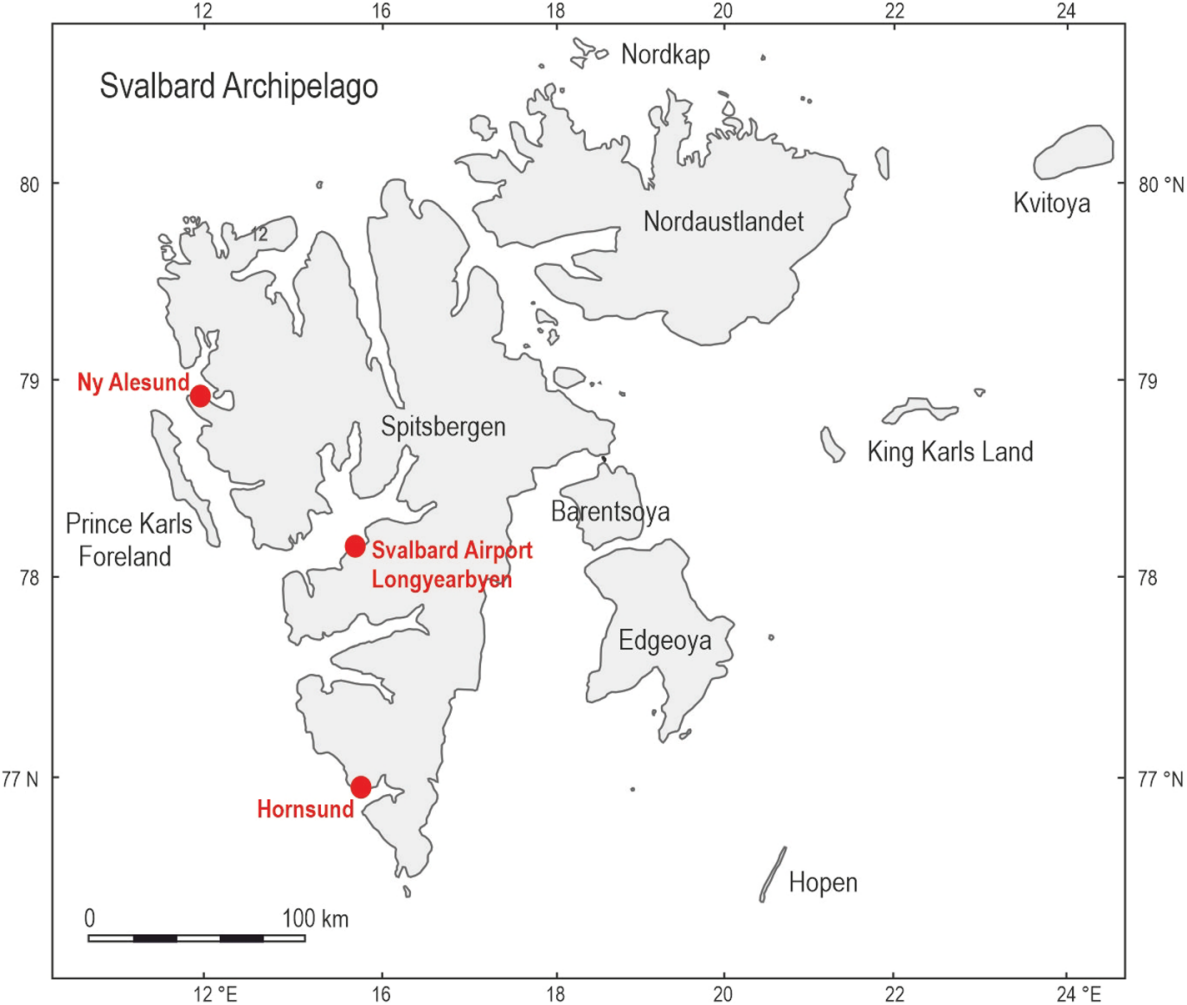
Location of the study area.

Ny-Ålesund is a coastal northwesternmost station. Svalbard Airport (Longyearbyen) represents the central and more continental part of the island, while Polish Polar Station at Hornsund is located on the northern coast of the southernmost Hornsund fjord in Spitsbergen (Fig. 1). The stations operate in accordance with operative measurement regulations and standards within the World Meteorological Organization and are respectively designated by the numbers 01007, 01008 and 01003^52^.

The NCEP/NCAR daily composites provided by the NOAA/ESRL Physical Sciences Laboratory, Boulder Colorado (https://psl.noaa.gov/data/composites/day/^53^), were used to document the synoptic conditions over the North Atlantic Ocean that determined the extreme pluviothermic seasons in Spitsbergen. The plots were generated for selected extreme values of SPEI. The following variables were used: sea level pressure, air temperature anomaly, 500 hPa geopotential height anomaly, omega index for 500 hPa explaining the vertical motion of air masses and precipitable water anomaly between 1000 and 500 hPa.

The methods used for distinguishing circulation types can be grouped into subjective, objective and hybrid, the third type being a mixture of the previous two. The usual ones are the Lamb classification for the British Isles^5^, and the Grosswetterlagen developed for Central Europe^54^. The objective classifications are based on a variety of statistical methods and are widely discussed^55–57^. Beck and Philipp^55^ who evaluated the performance of several classification schemes, found no superior, universal method and recommended a custom-designed approach. The influence of the regional atmospheric circulation on SPEI values in Svalbard was assessed using the catalogue of circulation types proposed by Niedźwiedź^58,59^, which has been widely used for studying the relationships between atmospheric circulation and climate change^60–62^. The catalogue was created manually based on German synoptic maps published in “Tägliche Wetterbericht (1950–1975), “Europäischer Wetterbericht 1976–2003” and after 2000 on the DWD Archives website (http://www.wetter3.de/Archiv/archiv_dwd.html).

This subjective classification includes 21 types described by capital letters indicating the direction of air advection identified as geostrophic wind using the isobar pattern at sea level. For example, the capital letters NE stand for north-easterly advection. The type of baric centre was identified based on the 1013 hPa sea level pressure threshold, which is an objective and accepted division for low/high air pressure. The anticyclonic and cyclonic types are denoted by “a” and “c”, respectively. The classification includes 16 advectional types and five following non-advective types: Cc—centre of a low, Ca—centre of a high, Ka—anticyclonic wedge or ridge of high pressure, Bc—trough of low pressure (different directions of airflow and frontal system in the axis of the trough) and unclassified type x—situations difficult to classify and pressure cols (the point of intersection of a trough and a ridge in the pressure pattern with no clear direction of flow). The method of Niedźwiedź^58,59^ is based on the classification of weather types for the British Isles by Lamb^63^, but was slightly modified—this is described in detail in Łupikasza et al.^2,64^. However, the objective method distinguishes only two non-advective type, so the reduction in detail applies to the following four types—Cc, Ca, Ka and Bc. In order to assess the long-term relationships between atmospheric circulation and SPEI, meridional and zonal circulation indices and their components were calculated separately for cyclonic and anticyclonic conditions on a monthly scale (Table 1) based on the circulation types occurrences. The components of meridional (Nci, Nai, Sci, Sai) and zonal circulation (Wci, Wai, Eci, Eai) were calculated as a sum of scores attributed to each circulation type (Table 1) and expressed as a percentage of days in a given month. Zonal and meridional circulation indices were calculated as differences between the appropriate components (Table 1). In the case of the zonal circulation index (W-Eci, W-Eai), the positive/negative difference indicates the domination of the western/eastern advection. In the case of the meridional circulation index (S-Nci, S-Nai), the positive/negative difference indicates the domination of the southern/northern advection. The theoretical range of meridional and zonal circulation indices is from -100% to + 100%, and the range of the components is from 0 to 100%.

**Table 1:** Description of circulation indices calculated based on circulation types frequency.

Circulation index	Description	Scores
Sci, Sai	Southern component of meridional circulation index for cyclonic (Sci) and anticyclonic (Sai) conditions. Frequency of air advection from the southern sector	Sci = nSc + nSEc*0.5 + nSWc*0.5 Sai = nSa + nSEa*0.5 + nSWa*0.5 n—number of occurences
Nci, Nai	Northern component of meridional circulation for cyclonic (Nci) and anticyclonic (Nai) conditions. Frequency of air advection from the northern sector	Nci = nNci + nNEc*0.5 + nNWc*0.5 Nai = nNa + nNEa*0.5 + nNWa*0.5
S-Nci, S-Nai	Meridional circulation index under cyclonic conditions (S-Nci) and anticyclonic conditions (S-Nai)	S-Nci = Sci – Nci S-Nai = Sai – Nai
Wci, Wai	Western component of zonal circulation for cyclonic (Wci) and anticyclonic (Wai) conditions. Frequency of air advection from the western sector	Wci = nWc + nSWc*0.5 + nNWc*0.5 Wai = nWa + nSWa*0.5 + nNWa*0.5
Eci, Eai	Eastern component of zonal circulation for cyclonic (Eci) and anticyclonic (Eai) conditions. Frequency of air advection from the eastern sector	Eci = nEc + nSEc*0.5 + nNEc*0.5 Eai = nEa + nEa*0.5 + NEa*0.5
W-Eci, W-Eai	Zonal circulation index under cyclonic conditions (W-Eci) and anticyclonic conditions (W-Eai)	W-Eci = Wci – Eci W-Eai = Wai – Eai

The standardized precipitation evapotranspiration index (SPEI) was calculated using daily observations of air temperature and precipitation from Ny-Ålesund, Longyearbyen (Svalbard Airport) and Hornsund. A climatological water balance time series for the 1979–2019 period was compiled from these meteorological variables. These observations were subjected to thorough quality control. Potential evapotranspiration (PET) was estimated using the Hamon method^65^ based on the daily air temperature and latitude of stations. The estimated water balance (difference between the sum of precipitation and PET) over different aggregation periods was normalized. In accordance with the literature review by Stagge et al.^45^, different distributions were tested, and their suitability was assessed based on Anderson–Darling and chi-square tests. The best fit was obtained for the generalized extreme value probability (GEV) distribution. Therefore the GEV distribution was fitted to the climatological water balance time series aggregated over a chosen period (annual, May–October, MAM, JJA, SON, and DJF). The same procedure was applied to all three stations, enabling comparison of the conditions between the stations. Trends in drought conditions were tested with the modified Mann–Kendall method for the autocorrelated data^66^. The slope of the trend was estimated using Sen’s method ^67^. These statistical methods are commonly used in analyses of changes in hydro-climatic conditions.

The SPEI classes proposed by Vicente-Serrano et al. (2010) were modified and applied: moderately wet 2 < SPEI ≤ 3; slightly wet 1 < SPEI ≤ 2; incipient wet spell 0.5 < SPEI ≤ 1; near normal −0.5 ≤ SPEI ≤ 0.5; incipient dry spell −0.5 > SPEI ≥ −1; slightly dry −1 > SPEI ≥ −2; moderately dry −2 > SPEI ≥ −3.

## Results and discussion

In the period analysed (1979–2019), normal conditions (−0.5 ≤ SPEI ≤ 0.5) occurred at an average annual frequency from 36.6% at Svalbard Airport to 39.0% at Ny-Ålesund and 41.5% at Hornsund. MAM was the season with the greatest variation in the frequency of near normal conditions between the three sites, with 53.7% at Svalbard Airport and 29.3% at Ny-Ålesund and Hornsund. Cases of drought (SPEI < −0.5) occurred most often in JJA, i.e. 34.1% at Ny-Ålesund and 36.6% each at the other two stations. SON was the wettest season (with SPEI > 0.5), with a frequency of 39.0% at Hornsund, 26.8% at Svalbard Airport and 36.6% at Ny-Ålesund, where a similar frequency of “wet conditions” occurred in MAM and JJA, and also in the period May–October (Table 2).

**Table 2:** Frequency of the drought index SPEI with extreme values in the seasons (annual, May–October and quarter seasons MAM, JJA, SON, DJF and monthly absolute extremes) at Ny-Ålesund, Svalbard Airport and Hornsund (Spitsbergen) in 1979–2019.

Ny Alesund	SPEI classes	Annual	May–Oct	MAM	JJA	SON	DJF	Monthly absolute extremes
		No of cases						
Moderately wet	2 < SPEI ≤ 3	0	1	0	1	1	2	
Slightly wet	1 < SPEI ≤ 2	8	4	8	4	5	4	
Incipient wet spell	0.5 < SPEI ≤ 1	5	10	7	10	9	6	
Near normal	0.5 ≥ SPEI ≥ −0.5	16	14	12	12	12	16	
Incipient dry spell	− 0.5 >SPEI ≥ − 1	4	4	7	7	8	5	
Slightly dry	− 1 > SPEI ≥ − 2	8	8	7	7	5	6	
Moderately dry	− 2 > SPEI ≥ − 3	0	0	0	0	1	1	
		% of cases						
Wet	SPEI > 0.5	31.7	36.6	36.6	36.6	36.6	30.0	
Near normal	0.5 ≥ SPEI ≥ −0.5	39.0	34.1	29.3	29.3	29.3	40.0	
Dry	SPEI < −0.5	29.3	29.3	34.1	34.1	34.1	30.0	
MAX value/year		1.88/2016	2.48/2000	1.63/1990	2.15/2013	2.10/2016	2.08/2006	2.82/ May 2014
MIN value/year		−1.99/1995	−1.97/1995	−1.99/2018	−2.00/1985	−2.29/1995	−2.20/2000	−3.00/ Apr. 2006

Svalbard Airport		No of cases						
Moderately wet	2 < SPEI ≤ 3	1	1	2	1	1	1	
Slightly wet	1 < SPEI ≤ 2	6	6	3	5	4	5	
Incipient wet spell	0.5 < SPEI ≤ 1	5	7	6	10	6	8	
Near normal	0.5 ≥ SPEI ≥ −0.5	15	17	22	10	19	13	
Incipient dry spell	− 0.5 >SPEI ≥ − 1	7	3	4	10	7	4	
Slightly dry	− 1 > SPEI ≥ − 2	7	7	2	4	3	9	
Moderately dry	− 2 > SPEI ≥ − 3	0	0	2	1	1	0	
		% of cases						
Wet	SPEI > 0.5	29.3	34.1	26.8	39.0	26.8	35.0	
near normal	0.5 ≥ SPEI ≥ −0.5	36.6	41.5	53.7	24.4	46.3	32.5	
Dry	SPEI < −0.5	34.1	24.4	19.5	36.6	26.8	32.5	
MAX value/year		2.33/1981	2.18/1981	2.49/1993	2.39/1981	2.76/2016	2.07/1996	2.84/ Apr. 1990
MIN value/year		−1.96/1998	−1.69/2009	−2.92/2006	−2.10/2007	−2.60/1995	−1.78/1987	−3.77/ Apr. 2006

Hornsund		No of cases						
Moderately wet	2 < SPEI ≤ 3	2	1	1	0	1	1	
Slightly wet	1 < SPEI ≤ 2	3	5	6	6	5	5	
Incipient wet spell	0.5 < SPEI ≤ 1	6	7	8	5	10	5	
Near normal	0.5 ≥ SPEI ≥ −0.5	17	13	12	15	12	17	
Incipient dry spell	− 0.5 >SPEI ≥ − 1	8	7	6	6	4	9	
Slightly dry	− 1 > SPEI ≥ − 2	3	7	6	9	8	3	
Moderately dry	− 2 > SPEI ≥ − 3	2	1	2	0	1	1	
		% of cases						
Wet	SPEI > 0.5	26.8	31.7	36.6	26.8	39.0	26.8	
Near normal	0.5 ≥ SPEI ≥ −0.5	41.5	31.7	29.3	36.6	29.3	41.5	
Dry	SPEI < −0.5	31.7	36.6	34.1	36.6	31.7	31.7	
MAX value/year		2.29/2016	2.00/1994	2.31/1982	1.92/1994	2.16/2016	2.46/1996	2.81/ Apr. 1982
MIN value/year		−2.35/2019	−2.13/1987	−2.15/2019	−1.77/2017	−2.07/1983	−2.64/1988	−2.96/ Apr. 2006

Trends in drought conditions are presented in Table 3. Statistically significant changes were obtained for various seasons, depending on the station. The directions of changes were the same for Ny-Ålesund and Hornsund. At these two stations (NyA, HOR), positive trends indicating wetter conditions dominate, while in Longyearbyen (LYR), negative trends are significant, indicating progressively drier conditions. Significant trends in the same direction at least at two stations were found in MAM, SON and DJF. The largest changes were found for autumn, where negative SPEI values occurred more often in the first part of the period and positive values dominated in the last 20 years. Trends in circulation indices could contribute to significant trends in seasonal SPEI. The wetting in DJF can be related to the increased frequency of air advection from the southern sector, particularly during cyclonic conditions (Sci) and decreased frequency of eastern advection under anticyclonic conditions (Eai). Drying in MAM and JJA could be linked to decreasing trends in meridional circulation due to the increased frequency of air advection from the northern sector. Wetting in SON agreed with significant negative trends in air advection from the eastern sector under the influence of cyclone (Eci, Table 4). All circulation indices used above to explain trends in wetting or drying were significantly correlated with SPEI. The relationships between SPEI and circulation indices for JJA and DJF are discussed further in this section.

**Table 3:** Trends in SPEI with modified Mann–Kendall method.

	SPEI (change per decade)		
	Ny-Ålesund (NYA)	Svalbard Airport (LYR)	Hornsund (HOR)
Annually	0.303	−0.272	0.332*
May–Oct	0.056	−0.365*	0.303*
MAM	−0.163	−0.360***	−0.228***
JJA	−0.257	−0.349**	−0.085
SON	0.314**	0.200	0.482***
DJF	0.380**	0.182	0.182**

Statistical significance: * ≤ 0.05, ** ≤ 0.01, *** ≤ 0.001.

**Table 4:** Trends (change per decade) in seasonal circulation indices.

Index	DJF	MAM	JJA	SON
S-Nci	1.12	−2.65*	−2.35*	−0.50
Sci	1.72**	0.19	−0.79	0.30
Nci	0.10	2.41***	1.50*	0.90
S-Nai	2.33***	−0.62	−3.01**	0.84
Sai	0.71	−0.03	−0.78	0.59
Nai	−1.34***	0.39	1.51*	−0.34
W-Eci	0.28	−0.53	0.42	3.46*
Wci	−0.80*	−0.61*	−0.45	0.69**
Eci	−1.10	−0.17	−0.67	−2.23**
W-Eai	1.15*	2.17*	0.13	0.79
Wai	−0.03	0.37	−0.30	0.34
Eai	−1.06*	−1.71	−0.53	−0.37

Statistical significance: * ≤ 0.05, ** ≤ 0.01, *** ≤ 0.001.

**Figure 2:**
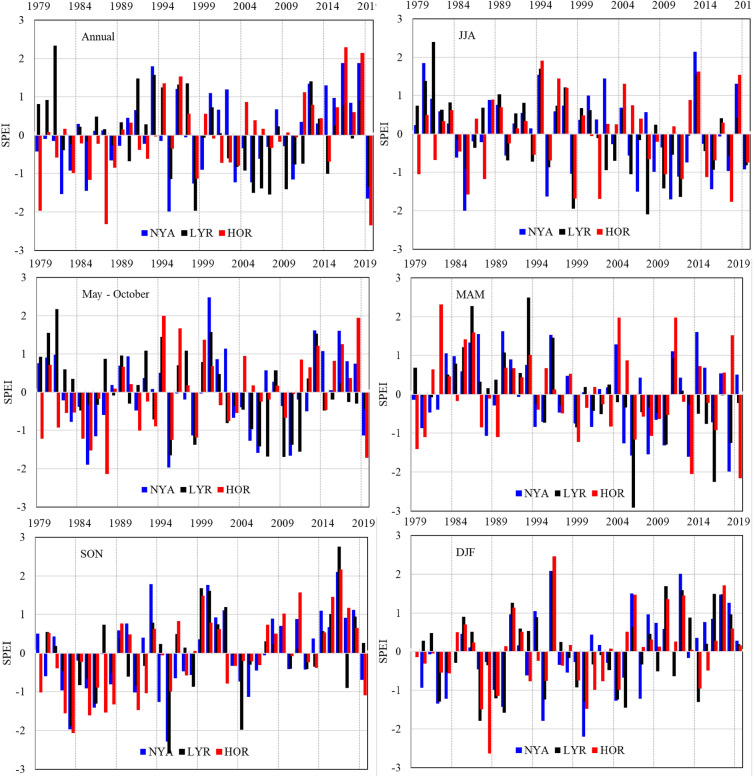
The Standardised Precipitation Evapotranspiration (SPEI) (annual, May–October and quarter seasons MAM, JJA, SON, and DJF) at Ny-Ålesund, Longyearbyen and Hornsund, Spitsbergen in 1979–2019. Drought classes (SPEI): moderately wet 2 < SPEI ≤ 3; slightly wet 1 < SPEI ≤ 2; incipient wet spell 0.5 < SPEI ≤ 1; near normal −0.5 ≤ SPEI ≤ 0.5; incipient dry spell −0.5 > SPEI ≥ −1; slightly dry −1 > SPEI ≥ −2; moderately dry −2 > SPEI ≥ −3.

The SPEI values were characteristic of interseasonal variability (Fig. 2). In a particular season, all the stations usually experienced the same conditions (dry or wet) but of various intensities. Seasons with extremely different conditions at the stations were rare, indicating the possible impact of large-scale atmospheric conditions on SPEI values and their modifications by local conditions around the stations.

In the period studied, it was possible to indicate several-year periods with the SPEI of the same sign (plus or minus), showing the dominance of drought or wet conditions. The long-term variability in the annual and half-year (May–October) SPEI values showed a prevalence of droughts in the 1980s and the first decade of the twenty-first century, while wet seasons were frequent in the 1990s and the second decade of the twenty-first century. In MAM and JJA, droughts were more frequent after 2000; the frequency of wet seasons increased in SON and DJF of the same period. The most remarkable changes in the scale of the entire research period were found in autumn, where negative SPEI values occurred more often in the first part of the period and positive values dominated in the last 20 years. Positive trends in SON and DJF SPEI were related to a significant increase in precipitation total which accompanied warming. In MAM and JJA decreasing trends in SPEI resulted from significant increase in air temperature and no trends in precipitation amount in the research period.

**Figure 3:**
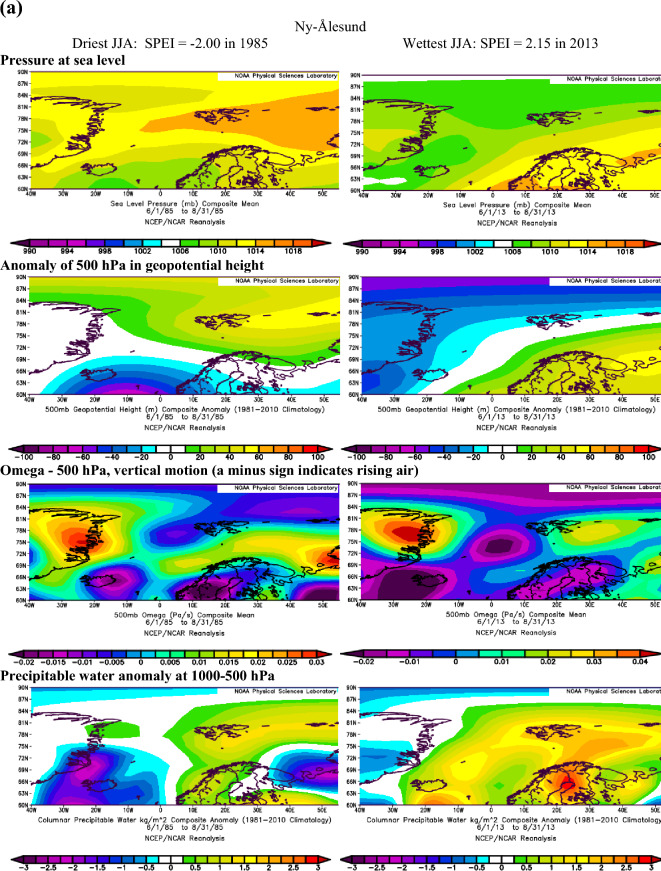

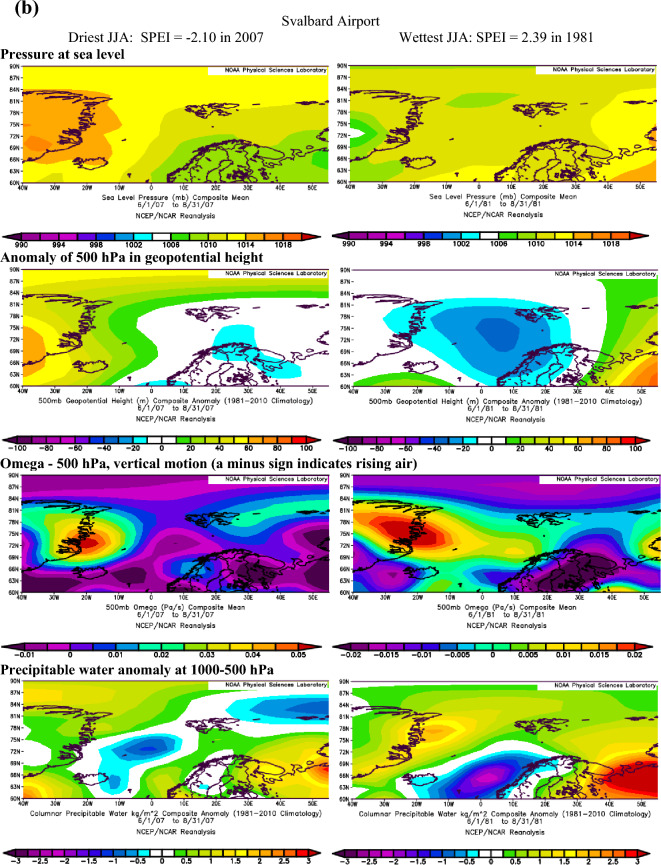

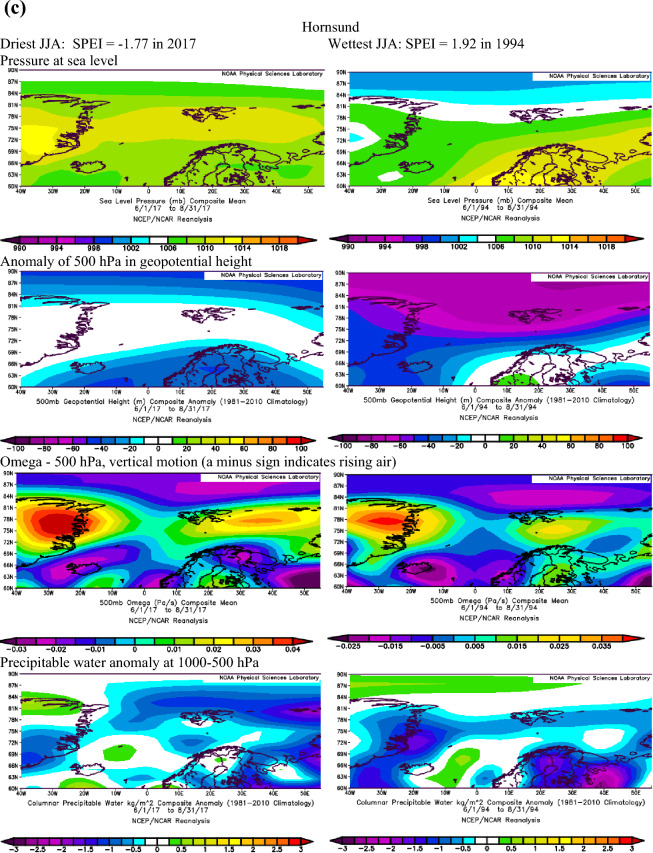
(**a**) The atmospheric conditions over the North Atlantic formed in JJA for the most extreme values of drought conditions at Ny-Ålesund, W Spitsbergen. Image provided by the NOAA/ESRL Physical Sciences Laboratory, Boulder Colorado from their Web site at http://psl.noaa.gov and downloaded on10 December 2021. (**b**) The atmospheric conditions over the North Atlantic formed in JJA for the most extreme values of drought conditions at Svalbard Airport, W Spitsbergen. Image provided by the NOAA/ESRL Physical Sciences Laboratory, Boulder Colorado from their Web site at http://psl.noaa.gov and downloaded on10 December 2021. (**c**) The atmospheric conditions over the North Atlantic formed in JJA for the most extreme values of drought conditions at Hornsund, W Spitsbergen. Image provided by the NOAA/ESRL Physical Sciences Laboratory, Boulder Colorado from their Web site at http://psl.noaa.gov and downloaded on 10 December 2021.

**Figure 4:**
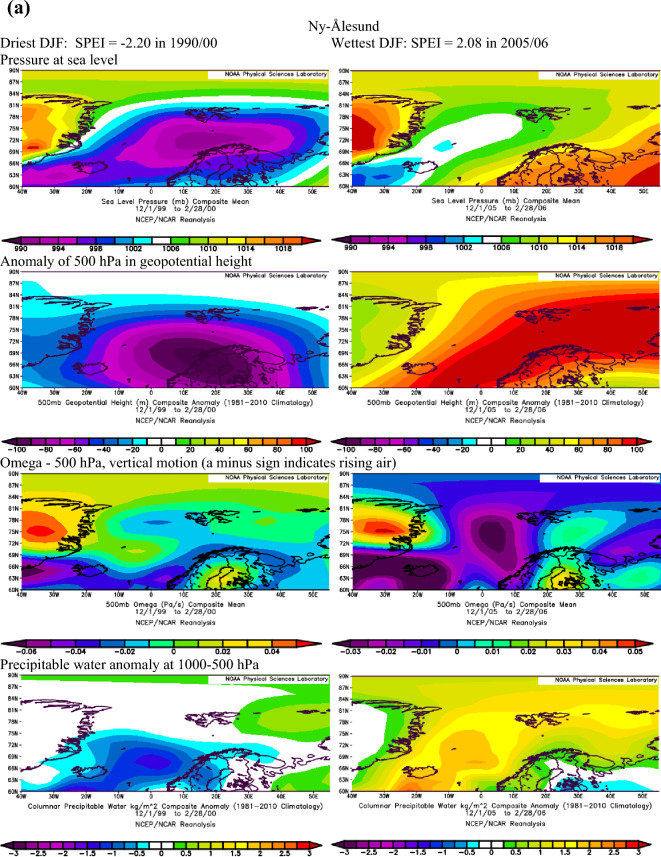

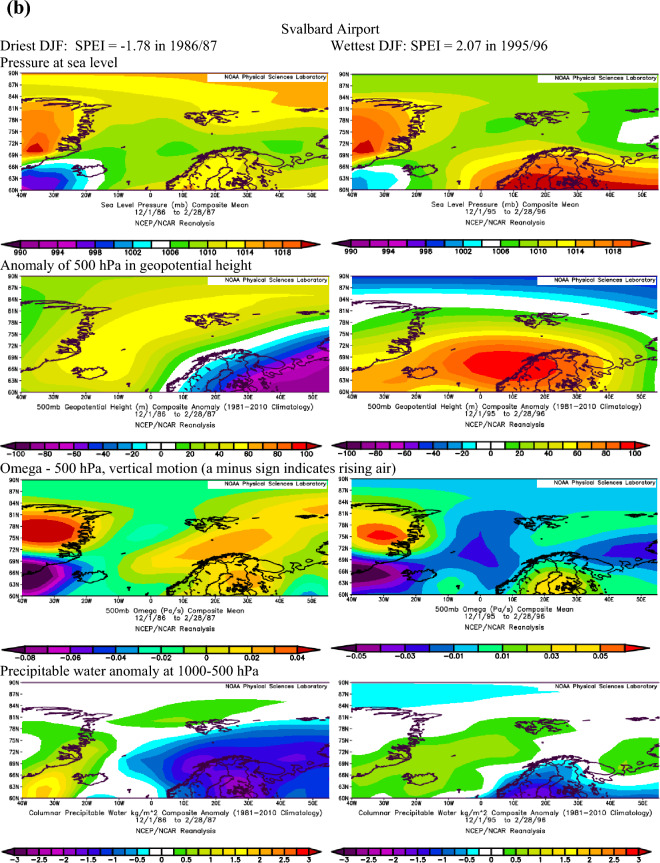

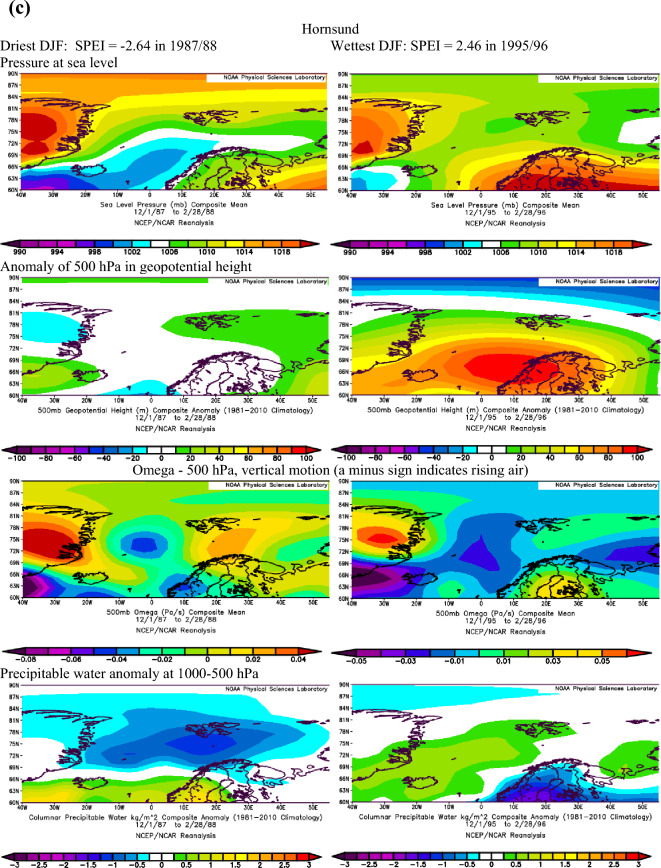
(**a**) The atmospheric conditions over the North Atlantic formed in winter (DJF) for the most extreme values of drought conditions at Ny-Ålesund, W Spitsbergen. Image provided by the NOAA/ESRL Physical Sciences Laboratory, Boulder Colorado from their website at http://psl.noaa.gov and downloaded on 22 January 2022. (**b**) The atmospheric conditions over the North Atlantic formed in winter (DJF) for the most extreme values of drought conditions at Svalbard Airport, W Spitsbergen. Image provided by the NOAA/ESRL Physical Sciences Laboratory, Boulder Colorado from their website at http://psl.noaa.gov and downloaded on 22 January 2022. (**c**) The atmospheric conditions over the North Atlantic formed in winter (DJF) for the most extreme values of drought conditions at Hornsund, W Spitsbergen. Image provided by the NOAA/ESRL Physical Sciences Laboratory, Boulder Colorado from their website at http://psl.noaa.gov and downloaded 22 on January 2022.

Extending the findings by Serreze et al.^40^ on conditions favouring the occurrence of extreme precipitation in the Arctic, we assume that dry conditions identified with the SPEI are also linked to the patterns of geopotential height and precipitable water over the Atlantic sector of the Arctic. The atmospheric conditions over the North Atlantic that occurred during months with the most extreme SPEI values in summer and winter at Ny-Ålesund, Svalbard Airport/Longyearbyen and Hornsund are shown in Figs. 3a–c and 4a–c.

Atmospheric conditions differed between the studied extremely wet and dry seasons and these differences also depended on the season. During the selected extremely wet seasons, anomalies of 500 hPa geopotential height over Svalbard were negative in summer (colder) and positive in winter (warmer), Omega 500 hPa were negative indicating rising air, and anomalies of PW were also positive. Such a pattern was found for almost each station with minor exceptions. The distribution of SLP in DJF indicated the advection of air from the south-western sector or the trough over the Spitsbergen, thus positive 500 hPa anomalies. During extremely wet summers the air advection from the SW sector was evident over the northern part of the North Atlantic; however, it was weakened over Spitsbergen, which resulted in a slightly increased impact of colder air from the western sector thus leading to negative anomalies of 500 hPa geopotential height. Nonetheless, in both seasons, DJF and JJA, the discussed extremely wet conditions were related to very high precipitation regardless of differences in air temperature. The conditions favouring the occurrence of extreme precipitation, giving rise to high SPEI values identified by Serreze et al.^40^ for short-term states of the atmosphere generally agree with conditions for high SPEI. A slightly less evident pattern for SPEI compared to that by Serreze et al.^40^ results from the contribution of not only precipitation but also air temperature to SPEI.

In the case of analysed extremely dry seasons, the anomalies of 500 hPa geopotential height were negative only at the Ny-Ålesund in both seasons (colder), did not differ from average conditions at Longyearbyen and Hornsund in JJA and were positive at Longyearbyen and Hornsund (warmer) in DJF. The distribution of SLP indicated an eastern advection of the air during anticyclonic conditions (in DJF, and in JJA only at LYR) or a high-pressure ridge over Spitsbergen (Ny-Ålesund and Hornsund in JJA). Such a pattern with dominant anticyclonic conditions usually blocks the intrusion of midlatitude lows and the related transport of wet air masses. The low-pressure zone is then shifted to the south and stretches along the trajectories of the atmospheric fronts moving zonally from Iceland towards Scandinavia^14^. The Omega-500 hPa was positive in JJA which indicated a weakening of convection during the studied extremely dry seasons, but took various signs depending on the station in DJF. The anomalies of PW were equal or slightly above 0 except for Hornsund in DJF. Weak anomalies of PW for the studied dry seasons may partly be related to the role of air temperature (not only precipitation) on the SPEI value. Generally, during selected extremely dry events, atmospheric conditions were more coherent in DJF than in JJA. Such complicated atmospheric conditions during the studied dry JJA seasons may indicate the role of local conditions in modifying precipitation amount and air temperature, thus impacting the development of droughts.

**Figure 5:**
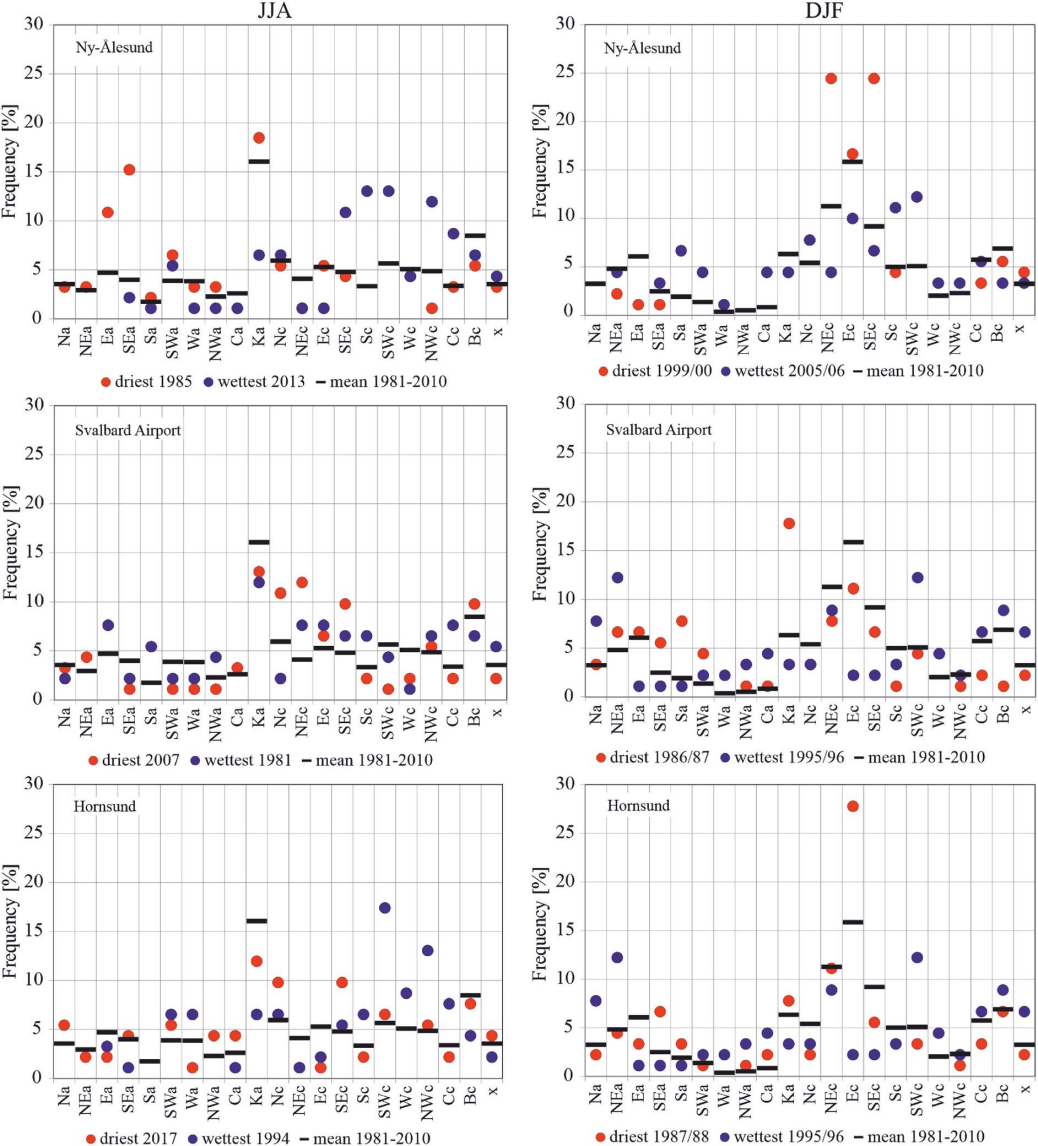
The frequency of atmospheric circulation types in the JJA and DJF seasons with the lowest and the highest values of SPEI vs the climatic normal 1981–2010.

Atmospheric conditions for extremely dry seasons show a large spatial diversity; thus the patterns conducive to droughts become less clear. Therefore, in the next step, the circulation types were analysed as decisive for the occurrence of extremely dry and wet conditions in JJA and DJF, when drought or abundant water are crucial for vegetation, ablation and melting of the active layer of permafrost (in JJA) or snowfall resources (in DJF) (Fig. 5).

The three stations are located at distances of more than 200 km from each other in the northern, central and southern parts of western Spitsbergen. In this area, the selected most extreme values of drought conditions developed under the influence of various circulation types. However, there were some similarities in the patterns of circulation type frequencies favouring extremely wet and dry conditions between Ny-Ålesund and Hornsund, both having more maritime climates than Longyearbyen. In the driest summer (JJA), in Ny-Ålesund anticyclonic circulation (a) with air advection from the SE dominated, followed by the centre of a high-pressure ridge (more stable atmosphere) over Svalbard (type Ka), which was also most frequent during the driest summers in Longyearbyen and at the Hornsund station. Moreover, during the driest summer in Longyearbyen the advection of cold, dry air from the north sector (N, NE) under the influence of cyclonic circulation was also frequent. Additionally, except for Ny Ålesund, the cyclonic circulation from SE was also frequent. During the studied summers, those days (with SEc type) were warmest compared to others with no precipitation at both stations. Cyclonic conditions (c) with air advection from S, SW and central cyclonic type (Cc) dominated during extremely wet JJA seasons all over Svalbard. Many papers indicate an increase in precipitation over Svalbard during air advection from the southern sector ^68–70^. Moreover, the high frequency of SEc and NWc types in Ny-Ålesund and cyclonic advection from the NW in Hornsund were also important in shaping the studied extremely wet summers (Fig. 5).

At Ny-Ålesund, the driest winter (DJF) distinguished with the high frequency of the cyclonic advection of cold, dry air from the NE and warmer and dry air from the SE which intensified evaporation. During the wettest DJF there were increased frequencies of cyclonic circulation (c) with air advection from both the S and SW. Regardless of the baric type, air masses from the S sector, particularly from the SW, are warmer and wetter than the Arctic air. The driest DJF in the central part of Svalbard was characteristic of a high frequency of high pressure ridge (type Ka) and anticyclonic circulation (a) from the S, SE, and SW. In the central and southern parts of Svalbard, during the wettest DJF, SWc type (the occurrence of precipitation) and the anticyclonic NEa type (low evaporation due to low temperatures) dominated (Fig. 5).

Next, Pearson correlation coefficients were calculated (Table 5) between the SPEI and zonal and meridional circulation indices, defined in Table 1, in order to recognise long-term relationships between all SPEI values (not only selected extreme seasons) and atmospheric circulation. In DJF the SPEI was strongest driven by zonal circulation during cyclonic conditions. The increase in air advection from the western sector favoured the wet conditions, while the eastern inflow of air favoured the occurrence of droughts. Zonal circulation explained about 30% of the variance in SPEI at Ny-Ålesund and Hornsund, while the relationships were weaker at Longyearbyen, located in more continental conditions. This finding supports the distribution of SLP during the studied extremely dry winter seasons (Fig. 4a–c, distribution of SLP). In DJF, situations such as these form over Svalbard when high pressure centres develop over Greenland and the central Arctic and reach the Barents Sea (advection from the eastern sector) which blocks the transport of moisture usually associated with the cyclonic advection of air masses from the SW sector.

**Table 5:** Pearson correlation coefficients between SPEI and circulation indices in DJF and JJA based on series detrended with first-degree polynomial.

Season	St	Zonal circulation indices and their components					
		W-Eci	Wci	Eci	W-Eai	Wai	Eai
DJF	NyA	0.589***	0.622***	−0.519***	0.063	0.432**	0.065
	LYR	0.434**	0.508***	−0.366*	−0.066	0.127	0.105
	HOR	0.554***	0.541***	−0.501***	0.037	0.163	0.011
JJA	NyA	0.466**	0.555***	−0.200	0.333*	−0.005	−0.408**
	LYR	0.534***	0.613***	−0.259	0.536***	0.251	***
	HOR	0.480**	0.567***	−0.212	0.601***	0.360*	−0.564***

Season	St	Meridional circulation indices and their components					
		N-Sci	Sci	Nci	N-Sai	Sai	Nai
DJF	NyA	0.270	0.075	−0.321*	0.000	0.188	0.190
	LYR	0.074	−0.058	−0.172	−0.194	−0.095	0.175
	HOR	0.335*	0.298	−0.182	−0.136	−0.018	0.173
JJA	NyA	0.481**	0.615***	−0.035	0.200	−0.114	−0.342*
	LYR	0.398**	0.608***	0.070	0.036	−0.236	−0.215
	HOR	0.539***	0.700***	−0.029	0.147	−0.243	−0.365*

*W-Ec*i zonal circulation index under cyclonic conditions, *W-Eai* zonal circulation index under anticyclonic conditions, *Wai* western component of the zonal circulation index under anticyclonic conditions, *Wci* western component of the zonal circulation index under cyclonic conditions, *Eci* eastern component of the zonal circulation index under cyclonic conditions, *Eai* eastern component of the zonal circulation index under anticyclonic conditions, meridional circulation indices: N-Sci , N-Sai, Ni, Nci, Nai, Si Sci, Sai—analogous to the western circulation index, statistical significance: * ≤ 0.05, ** ≤ 0.01, *** ≤ 0.001.

In JJA the relationships between circulation indices and SPEI were more complicated. Western component of zonal circulation, i.e. air advection from the western sector during cyclonic conditions enhanced wetting (positive correlation between SPEI and Wci). The Wci index explained between 30 and 40% of the variance in SPEI. Interestingly, the eastern component was significantly negatively correlated with SPEI only during anticyclonic conditions, the strongest at Hornsund and the weakest at Ny Ålesund (Table 5). This means that increased air advection from the eastern sector during anticyclonic conditions favoured the occurrence of summer droughts due to very low precipitation during such advection which was previously found for Hornsund^40^.

Considering meridional circulation in JJA, the southern component during cyclonic conditions (Sci) and the northern component during anticyclone (Nai) were both correlated with SPEI, meaning that a decrease/increase in the frequency of air inflow from S during cyclone led to dryer/wetter conditions and from N during anticyclone led to opposite reaction. However, the impact of southern air advection that explained between 40 and 50% of the variance in SPEI was stronger (Table 5). Increased frequency of air advection from both the S sector during a cyclone favoured wetter conditions due to high precipitation during cyclonic S air advection (high Sci values) and increased frequency of air inflow from N sector during the anticyclone favoured dry conditions due to low precipitation (high Nai values).

Although the difference in precipitable water (PW) between the selected driest and wettest seasons was not very clear, the long-term pattern of the variables in consecutive seasons between 1979 and 2019 indicates strong relationships between the SPEI drought index and precipitable water (PW) anomalies. The precipitable water anomalies during dry conditions at Ny-Ålesund differed from those at Longyearbyen and Hornsund both having similar patterns of drought-favourable atmospheric conditions.

**Figure 6:**
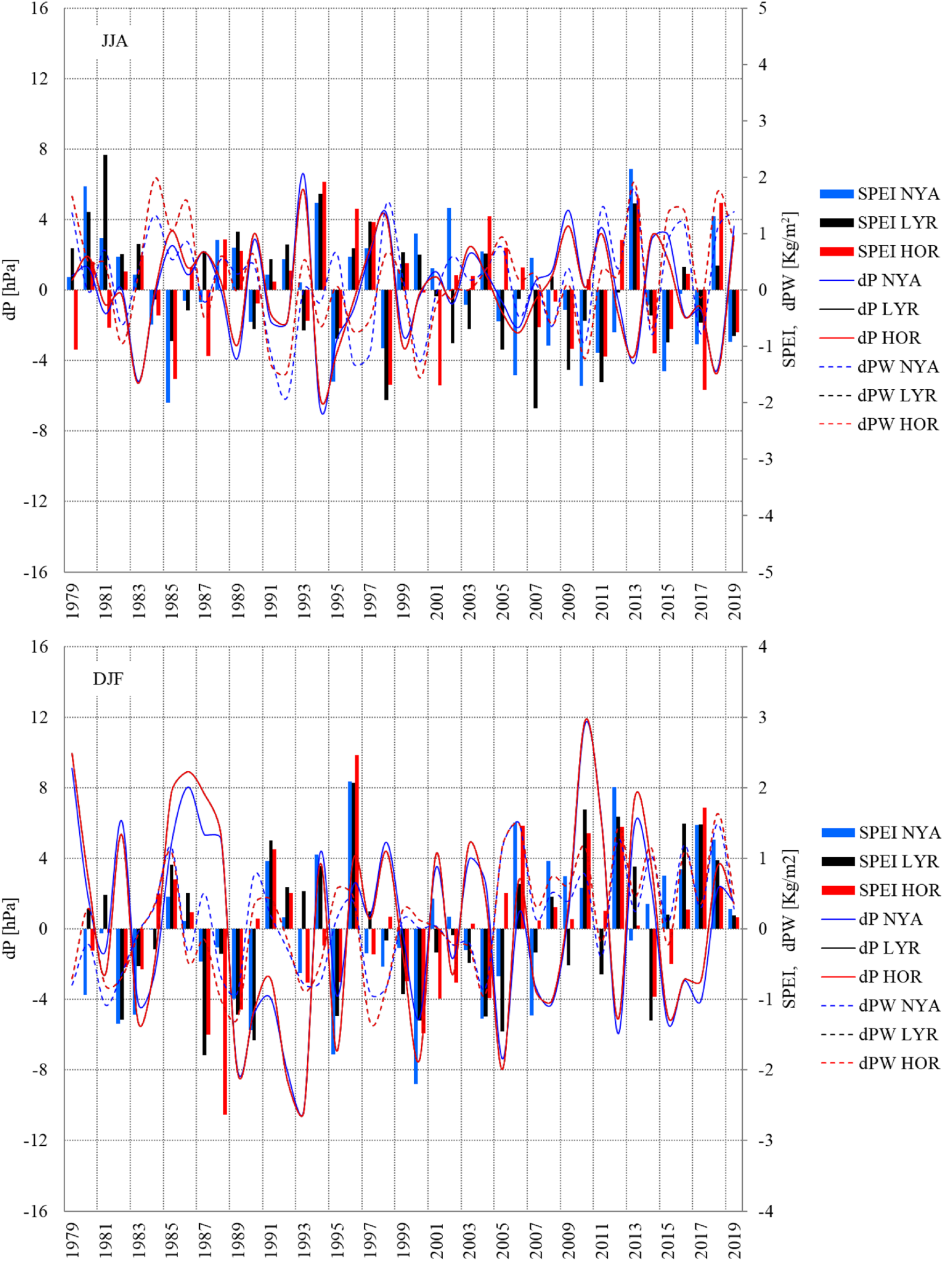
SPEI in summer (JJA) and winter (DJF) and anomalies of mean atmospheric pressure (dP) and precipitable water (dPW) vs the climatic normal 1981–2010 at Ny-Ålesund (NYA), Longyearbyen (LYR) and Hornsund (HOR). Image provided by the NOAA/ESRL Physical Sciences Laboratory, Boulder Colorado from their website at http://psl.noaa.gov and downloaded on 28 January 2022.

During most of the research period, dry and wet seasons occurred alternatively as mesoscale phenomena appearing simultaneously over the entire area of Spitsbergen in particular years (Figs. 2 and 6). In certain years, however, opposite conditions occurred in the same year, e.g., drought in the north and wet conditions in the south, or vice versa. In 1979–2019, the months with uniformly wet or dry conditions constituted 39% to 41.5% of the cases for both seasons, while the opposite conditions were represented by one-third of the cases (24.4%-26.8%) (Table 6).

**Table 6:** Years with the largest SPEI differences between Ny-Ålesund, Longyearbyen and Hornsund in July, summer and winter.

SPEI JULY				SPEI JJA				SPEI DJF			
Year	NyA	LYR	HOR	Year	NyA	LYR	HOR	Year	NyA	LYR	HOR
1986	−0.54	−0.28	1.07	1979	0.23	0.74	−1.05	1980	−0.93	0.28	−0.31
1991	0.94	−0.03	−0.66	1987	−0.22	0.68	−1.17	1993	−0.62	0.53	−0.77
1992	0.34	−0.10	−0.99	2000	1.00	0.63	−0.05	1994	1.05	0.89	−0.23
2001	−0.65	0.24	−0.86	2001	0.38	−0.11	−1.69	2001	0.43	−0.33	−0.99
2002	1.27	−0.78	−0.42	2002	1.45	−0.94	0.26	2005	−0.67	−1.45	0.51
2007	1.03	−1.34	−0.75	2005	−0.56	−1.05	0.75	2007	−1.22	−0.33	0.12
2008	−0.59	0.72	−0.35	2006	−1.51	−0.16	0.4	2009	0.75	−0.51	0.13
2010	−1.24	−0.26	0.77	2007	0.57	−2.1	−0.66	2013	−0.16	0.88	0.04
2012	−0.74	0.23	0.32	2008	−0.99	0.24	−0.21	2014	0.35	−1.3	−0.96
2017	0.38	0.16	−0.85	2010	−1.7	−0.54	0.2	2015	0.76	0.2	−0.5
				2012	−0.74	−0.04	0.89				

For two reasons special attention was focused on SPEI values in the summer (JJA) and its individual months. It was assumed that the analysis of atmospheric conditions on a monthly scale would help to explain the spatial variations in wet/dry conditions. Moreover, analysis on a monthly scale enables the relationships between the SPEI and tundra vegetation to be described. To this end, July was selected, when the intense development of vegetation is not limited by snow cover, as it still is in June.

**Figure 7:**
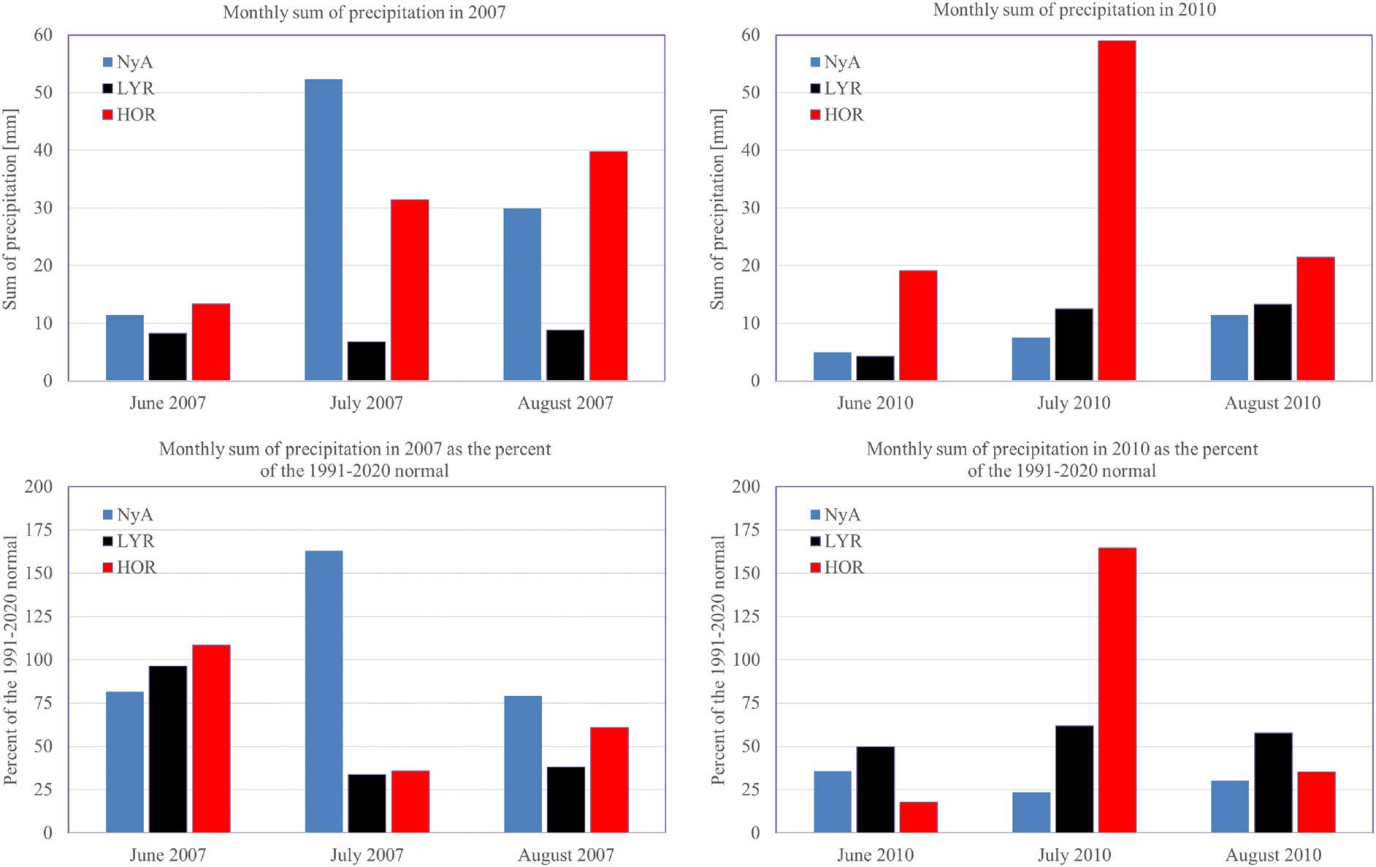
Monthly precipitation in the summer seasons of 2007 and 2010 at Ny-Ålesund (NyA), Longyearbyen (LYR) and Hornsund (HOR) vs normal of the 1991–2020.

The summers of 2007 and 2010 illustrate the contrasts in wet/dry conditions over Svalbard. In the summer of 2007, particularly in July, the northern part of Svalbard (Ny-Ålesund) with slightly wet conditions (SPEI = 1.03) contrasted strongly with the drought that occurred in central (Longyearbyen, SPEI: -1.34, slightly dry) and south-western Svalbard (Hornsund, SPEI = −0.75). In July 2010, the opposite situation prevailed: drought in the north (Ny-Ålesund, SPEI = −1.24) and an incipient wet spell in the vicinity of Hornsund (SPEI = 0.77) and near normal conditions in Longyearbyen (SPEI = −0.26). Figure 7 illustrates the precipitation differences between the stations in the summer seasons of 2007 and 2010. Contrasts of SPEI in July 2007 and 2010 were due to large differences in precipitation totals in the north compared to the south of Svalbard. The total July precipitation at Ny-Ålesund in 2007 exceeded 52.3 mm i.e. 160% of the 1991–2020 average, while at Longyearbyen and Hornsund, the precipitation totals reached 6.8 mm and 16.1 mm, which was only 33–35% of the average. In 2010, the situation was the opposite, at Hornsund the total July precipitation was 164% of the climatic norm (59 mm), at Longyearbyen it was almost 62% (12.5 mm), and at Ny-Ålesund it was only around one-quarter of the norm (23.4%, i.e.7.5 mm), calculated as the average for the period 1991–2020.

**Figure 8:**
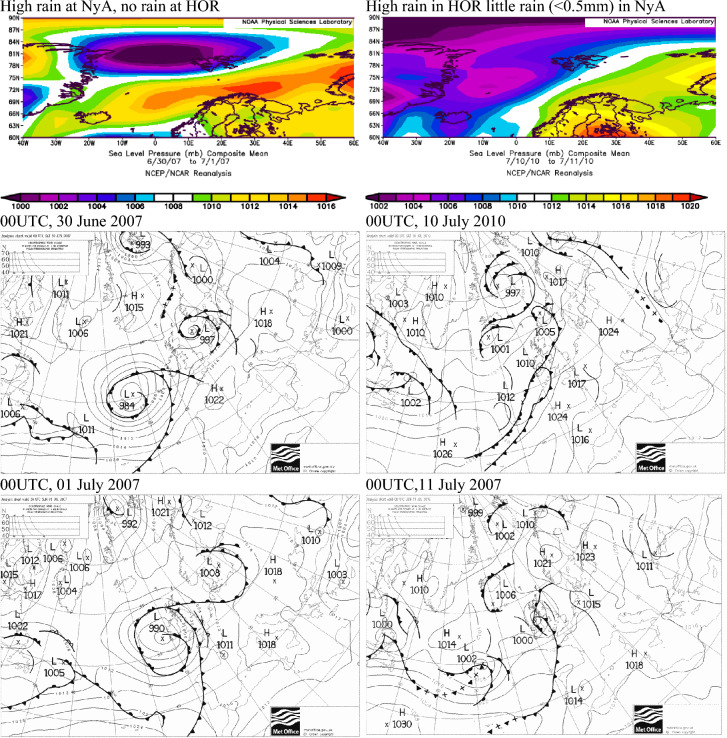
Sea Level Pressure over the North Atlantic on days with contrasting precipitation conditions between Ny-Ålesund, Svalbard Airport and Hornsund, 30.06-01.07. 2007 (precipitation in NyA, no precipitation in LYR and HOR), July 09, 2010 (precipitation in HOR, no precipitation in NyA). Image provided by the NOAA/ESRL Physical Sciences Laboratory, Boulder Colorado from their website at http://psl.noaa.gov and downloaded 11.02.2022. Synoptic maps for selected days with high precipitation at Spitsbergen (https://wetterzentrale.de/).

Atmospheric conditions during months with spatially contrasting SPEI conditions in the summers of July 2007 and 2010 did not differ over Svalbard because wet July in 2007 at Ny-Ålesund and in 2010 at Hornsund both developed due to 2 days long precipitation events—July 1–2, 2007 and July 10–11, 2010. On these days, the other stations did not experience precipitation at all or experienced very low precipitation. The distribution of SLP during these two-day periods and related synoptic charts show that in both cases, there were large gradients of SLP over Svalbard and atmospheric fronts related to low-pressure systems (Fig. 8). Thus at the beginning of July 2007, Ny-Ålesund was under the influence of low-pressure system with its centre located between northern Greenland and the northern part of Svalbard and a cold front that induced high precipitation in Ny-Ålesund at the beginning of July, while, at the same time, Hornsund was under the impact of the increased SLP related to a high-pressure ridge with its axis located between Svalbard and Scandinavian Peninsula (Fig. 8). The same front caused increased precipitation in Hornsund one day before i.e. at the end of June, which, however, was two times lower compared to Ny-Ålesund precipitation one day later. In 2010, high precipitation at Hornsund between 10 and 11 July were due to a small low-pressure system with its centre located south-west of Spitsbergen which induced precipitation in Hornsund but at other stations. Synoptic charts also revealed the stationary front located close to the southern Spitsbergen on July 10, 2010 that passed over Hornsund between July 10 and 11 and disappeared on the next day (Fig. 8). The studied contrasting dry/wet conditions were related to a specific synoptic situations with large gradients of SLP over or near Svalbard and possibly local conditions. High SPEI values indeed can be related to one-day precipitation events, therefore in order to identify atmospheric conditions conducive to drought, sub-monthly studies based on a selected set of dry days should be further studied.

## Conclusions

This paper discusses long-term variability and trends in drought index SPEI and related patterns of selected atmospheric variables for extreme, i.e. wettest and driest periods, on West Spitsbergen Island (Svalbard) in the period 1979–2019. Moreover, the relationships between SPEI values and atmospheric circulation described by circulation types and indices were studied. The analyses indicated that the average annual frequency of normal conditions (−0.5 ≤ SPEI ≤ 0.5) ranged from 36.6% at Svalbard Airport to 39.0% at Ny-Ålesund and 41.5% at Hornsund. MAM was the season with the greatest variation in the frequency of near normal conditions between the three sites, with 53.7% at Svalbard Airport and 29.3% at Ny-Ålesund and Hornsund. Cases of drought (SPEI < −0.5) occurred most often in JJA, i.e. 34.1% at Ny-Ålesund and 36.6% at each of the other two stations. SON was the wettest season (with SPEI > 0.5), with a 39.0% frequency at Hornsund, 26.8% at Svalbard Airport and 36.6% at Ny-Ålesund, where a similar frequency of “wet conditions” occurred in MAM and JJA, but also during the May–October period.

During this time, there were several years-long periods with SPEI of the same sign (plus or minus), indicating the dominance of drought or wet conditions. The long-term variability in the annual and half-year (May–October) SPEI values showed the prevalence of drought in the 1980s and in the first decade of the twenty-first century, whereas wet seasons were frequent in the 1990s and in the second decade of the twenty-first century. The seasonal SPEIs were characteristic of great interannual variability. In MAM and JJA, periods of drought were more frequent after 2000; in SON and DJF during the same period, the frequency of wet seasons increased. The most remarkable changes in the scale of the entire research period occurred in autumn, where negative SPEI values occurred more often in the first part of the period, whereas positive ones were dominant in the last 20 years.

During most of the periods analysed, dry and wet conditions occurred alternately as mesoscale phenomena appearing simultaneously over the whole of Spitsbergen in particular years. Extreme conditions occurred in different years at each station. In the NW part of Spitsbergen (Ny-Ålesund), the driest summer was in 1998 (SPEI −2.00), while that of 2013 was extremely wet (SPEI 2.15). In central Spitsbergen (Svalbard Airport/Longyearbyen), extreme summers occurred in 2007 with a drought index of −2.10 and in 1981 with the highest recorded SPEI value of 2.39. At Hornsund, representative of southern Spitsbergen, the driest summer season (JJA) was in 2017 (SPEI −1.77), and the wettest JJA occurred in 1994 (SPEI 1.92). At Ny-Ålesund, the driest winter (DJF) occurred in 1999/00 (SPEI −2.20), whereas the 2005/06 winter season was extremely wet. At Longyearbyen, the extreme seasons were 1986/87 (SPEI −1.78) and 1995/96 (SPEI 2.07). In the south of Spitsbergen, at Hornsund, extremely wet winters overlapped with those at Longyearbyen (1995/96—SPEI 2.07). During the driest winter of 1987/88, SPEI reached −2.64.

Extreme precipitation events and studied extreme wet conditions expressed by high, positive SPEI values both depended on the 500 hPa geopotential height and precipitable water anomalies, as determined by the baric field over the North Atlantic. In contrast, the selected extremely dry periods expressed by negative SPEI values were related to high pressure systems centred over Greenland and the central Arctic or high-pressure ridge over Spitsbergen that induced advection from the eastern sector and blocked the transport of moisture associated with the cyclonic advection of air masses from the SW sector. In summer, the studied dry conditions were also associated with ridges of high pressure or an extended area of increased pressure between the Greenland Sea and the Barents Sea.

The state of the atmosphere during extremely dry conditions indicates that anticyclonic conditions, particularly the Ka type, and air advection from the NE–E–SE sector and negative precipitable water anomalies, are decisive for dry conditions, in contrast to wet conditions, which are driven by positive precipitable water anomalies and cyclonic conditions with air advection from SW sector.

Climate models for Svalbard indicate an increase in precipitation for all seasons, with the highest and most statistically significant increase in the north–east and the smallest changes in the south–west^68^. This means that wetting trends expressed by positive SPEI values are generally projected to be observed, However, reconstruction of the baric field and atmospheric circulation will lead to changes in the spatial distribution of the drought index.

## Data Availability

The data that support the findings of this study are available from the following resources: NOAA/ESRL Physical Sciences Laboratory, Boulder Colorado from their Web site at http://psl.noaa.gov; Norwegian Centre for Climate services (https://seklima.met.no); Data Publisher for Earth & Environmental Science PANGAEA https://doi.org/10.1594/PANGAEA.909042; Calendar of Circulation Types for Spitsbergen—file downloaded from the Silesian University Website https://us.edu.pl/instytut/inoz/kalendarz-typow-cyrkulacji/.
